# CD36 is Associated With the Development of Coronary Artery Lesions in Patients With Kawasaki Disease

**DOI:** 10.3389/fimmu.2022.790095

**Published:** 2022-01-27

**Authors:** Mindy Ming-Huey Guo, Ying-Hsien Huang, Feng-Sheng Wang, Ling-Sai Chang, Kuang-Den Chen, Ho-Chang Kuo

**Affiliations:** ^1^ Department of Pediatrics, Kaohsiung Chang Gung Memorial Hospital and Chang Gung University College of Medicine, Kaohsiung, Taiwan; ^2^ Kawasaki Disease Center, Kaohsiung Chang Gung Memorial Hospital and Chang Gung University College of Medicine, Kaohsiung, Taiwan; ^3^ Graduate Institute of Clinical Medical Sciences, Chang Gung University College of Medicine, Taoyuan, Taiwan; ^4^ Department of Medical Research, Kaohsiung Chang Gung Memorial Hospital, Chang Gung University College of Medicine, Kaohsiung, Taiwan; ^5^ Center for Laboratory Animals, Kaohsiung Chang Gung Memorial Hospital, Chang Gung University College of Medicine, Kaohsiung, Taiwan; ^6^ Institute for Translational Research in Biomedicine, Liver Transplantation Center and Department of Surgery, Kaohsiung Chang Gung Memorial Hospital and Chang Gung University College of Medicine, Kaohsiung, Taiwan; ^7^ Department of Respiratory Therapy, Kaohsiung Chang Gung Memorial Hospital, Kaohsiung, Taiwan

**Keywords:** Kawasaki disease, coronary artery lesions, CD36, AIM2, macrophage

## Abstract

Kawasaki disease (KD) is an autoimmune-like vasculitis of childhood involving the coronary arteries. Macrophages require scavenger receptors such as CD36 to effectively clear cellular debris and induce self-tolerance. In this study, we hypothesized that CD36 plays an important role in the immunopathogenesis of KD, by aiding in the clearance of plasma mitochondrial DNA, and by amplifying the immune response by activating the inflammasome pathway *via* AIM2. Fifty-two healthy controls, 52 febrile controls, and 102 KD patients were recruited for RT-PCR of target mRNA expression and plasma mitochondrial DNA. Blood samples were obtained 24 hours prior and 21 days after the administration of intravenous immunoglobulin (IVIG) therapy. Patients with acute KD had higher plasma levels of cell-free mitochondrial DNA (ND1, ND4, and COX1), and higher mRNA expressions of CD36 and AIM2 when compared to both healthy and febrile controls. A greater decrease in both CD36 and AIM2 mRNA expression after IVIG therapy was associated with the development of coronary artery lesions. Coronary artery lesions were associated with a larger decrease of CD36 expression following IVIG therapy, which may indicate that prolonged expression of the scavenger receptor may have a protective effect against the development of coronary artery lesions in KD.

## Introduction

Kawasaki disease (KD) is the most common cause of acquired heart disease during childhood, especially in Asian countries like Japan and Taiwan (1, 2). Characteristic features of KD include fever lasting for at least 5 days, as well as at least four out of five of the following symptoms: erythema of the oral mucosa (fissured lips, strawberry tongue), bilateral non-suppurative conjunctivitis, lymphadenopathy, edema or erythema of the hands or feet, and a polymorphous rash (3). KD primarily involves inflammation of medium-sized vessels and can result in multiple cardiovascular sequelae, most commonly coronary artery aneurysms, and myocardial ischemia or infarction later in life (4).

Activated macrophages are critical to the development of coronary artery lesions in KD and produce such inflammatory cytokines as tumor necrosis factor-α and vascular endothelial growth factor, both of which further amplify the inflammatory response. They also produce proteases, including matrix metalloproteinase-2 (MMP-2) and MMP-9, which degrade the elastin fibers within the arterial wall, leading to coronary artery dilatation and aneurysms (5). Activated macrophages can be categorized as “classically-activated” M1 macrophages, which are mostly responsible for inflammatory and anti-microbial responses, and “alternatively-activated” M2 macrophages, which are considered immune-modulatory (6). In our previous research we have found that patients with Kawasaki disease had higher levels both M1 related cytokine TNF-α (7), and the M2 related cytokines IL-6, IL-4 and IL-13 in the acute phase of Kawasaki disease, which decreased after IVIG therapy (8, 9). These findings seem to suggest that both M1 and M2 macrophages are activated in the acute stage of KD. In fact, in a subsequent paper we have published which examined the expression of M1 and M2 macrophage surfaces markers, we found that, in the acute phase of KD, a higher percentage of M2 markers showed increased mRNA expression (10 out of the 15 M2 markers surveyed) when compared to M1 markers (2 out of the 10 M1 markers surveyed). Of particular interest, CD36, a M2 marker, has been extensively linked to the development of vascular disease and was found to have increased mRNA expression during the acute phase of KD, but decreased after IVIG therapy (10).

CD36 is a scavenger receptor expressed primarily on macrophages, but also expressed on a variety of cells, such as adipocytes, platelets, erythrocytes, endothelial cells, and muscle cells (11). Macrophages require scavenger receptors like CD36 for the phagocytosis of endogenous ligands, including oxidized phospholipids, lipoproteins, and exogenous ligands like lipoteichoic acid, as well as aid in the clearance of cellular debris from apoptosis and the development of self-tolerance (12). For example, in atherosclerosis, the uptake of oxidized low-density lipoproteins by CD36 increases actin polymerization, resulting in increased vascular inflammation caused by accumulation of macrophages within vessel walls (13). Increased expression of CD36 can also be induced by a high-fat diet. In an animal model of non-alcoholic liver disease, a high-fat diet induced increased mRNA expression in wild type mice, which was then ameliorated by increased expression of micro-RNA 29 (miR-29a) in transgenic mice. Transfection of HepG2 cells with a miR-29a mimic also decreased the expression of CD36 (14). CD36 also interacts with cell surface toll-like receptor (TLR) heterodimers TLR4/6 and TLR2/6 and aids in the endocytosis of endogenous oxidized LDL and β-amyloid, as well as exogenous lipoteichoic acid and mycoplasma macrophage-activating lipopeptide-2 (15, 16).

In our previous research, we found that patients with KD exhibited increased expression of almost all TLRs, with the exception of TLR3 and TLR7 in the acute phase of disease, both of which decreased after IVIG therapy (17). Toll-like receptors are membrane receptors that aid the innate immune system in recognizing both pathogen-associated molecular patterns (PAMPs) that originate from infectious pathogens and damage-associated molecular patterns (DAMPs) released by dying cells (18). Of note, TLR2, 4 and 6, which are expressed on the cell surface and interact with CD36 to aid in the endocytosis of extracellular lipoproteins and cellular debris, were all found to have increased expression in acute KD, which decreased following IVIG therapy (17). Furthermore, TLR9, another TLR found to have increased expression in the acute phase of KD (17), is expressed within the endosomal compartments and recognizes the CpG motif within DNA molecules, further propagating the immune response *via* activation of the inflammasome pathway (19).

Auto-antigens, including plasma mitochondrial DNA, are released by dying cells (20) and are a particularly potent inflammatory trigger. Unlike nuclear DNA, mitochondrial DNA contains a high percentage of unmethylated CpG motifs that are recognized and cleared by endosomal TLR9 (21), leading to the production of pro-inflammatory IL-1β, IL-18, and other products of the inflammasome pathway *via* AIM2 and NLRP3 (19). Mitochondrial DNA has been found to provoke autoimmune-like symptoms in animal models of systemic lupus erythematosus and rheumatic arthritis. In an animal model for lupus, researchers found that increased oxidative stress increased mitochondrial permeability and the release of mitochondrial DNA, as well as lupus-like symptoms (22). Similarly, in a mouse model of rheumatoid arthritis, intra-articular injection of mitochondrial DNA induced arthritis and the production of inflammatory tumor necrosis factor alpha *in vivo*, whereas the injection of nuclear DNA did not have the same effect (23). Given that KD has also been described as an autoimmune-like vasculitis, mitochondrial DNA may also be a possible trigger in the development of KD.

In this study, we hypothesized that CD36 also plays an important role in the immunopathogenesis of KD, first by aiding in the clearance of cellular debris and then by amplifying the immune response by activating the inflammasome pathway. Herein, we aimed to test whether active KD was associated with increased cellular debris burden by testing patient samples for plasma mitochondrial DNA. We also examined CD36 expression and interferon-inducible protein AIM2, an inflammasome receptor protein in KD patients with and without coronary artery lesions.

## Methods

### Subject Recruitment

We enrolled a total of 214 cases in this study, with 18 KD patients, 18 healthy controls (HC), and 18 febrile controls (FC) recruited for Human Transcriptome array analysis. In addition, a second cohort consisting of 52 healthy controls, 52 febrile controls, and 102 patients with KD was recruited for further confirmation of target mRNA expression and detection of plasma mitochondrial DNA. All KD patients recruited for this study met the criteria set forth by the American Heart Association, including having a fever lasting for more than five days and at least four out of five of the following clinical criteria: non-suppurative conjunctivitis, oral mucosal erythema, cervical lymphadenopathy, skin rash, and edema or erythema of the extremities (3). All patients received at least one dose of intravenous immunoglobulin (IVIG, 2g/kg/dose) infused over 12 hours according to current practice guidelines (3).The IVIG products used in this study were obtained from the Taiwan Blood Services Foundation and contained human immunoglobulin G (IgG) which was extracted from large pools of human plasma acquired from voluntary donors. IVIG resistance was defined as persistent fever 48 hours after completion of IVIG infusion. Patients with IVIG resistance were then given a second dose of IVIG (2g/kg/dose). Blood samples were obtained from KD patients within 24 hours prior to IVIG therapy (KD1 group) and then at least 21 days after IVIG therapy (KD2 group). Echocardiography was performed at the following time points: at the time of diagnosis, and one week, one month and six months thereafter. Patients who had coronary artery dilatation or ectasia that resolved within the first 4 weeks after diagnosis were not considered as having persistent coronary artery lesions. We defined the presence of coronary artery lesions (CAL) as the internal diameter of the coronary arteries having a z-score of ≥ 2.5 or if the absolute measurement of the coronary arteries were ≥ 3 mm (if younger than 5 years old) or ≥ 4 mm (if older than 5 years old) (24, 25). Informed consent was obtained from the parents or guardians of all patients included in this study. The studies involving human participants were reviewed and approved by the Internal Review Board of Chang Gung Memorial Hospital.

All blood samples obtained from KD patients and both healthy and febrile controls were first centrifuged. After being centrifuges, leukocytes were removed from the buffy coat layer for further mRNA extraction. Total RNA was then extracted using a commercial kit (mirVana™ miRNA Isolation Kit, Catalog number: AM1560, Life Technologies, Carlsbad, CA), with all RNA samples achieving a RIN (RNA integral number) of at least 7. We also extracted circulating DNA from a fixed volume of plasma (200 μL) from each sample according to the manufacturer’s instructions (QIAamp Blood Mini Kit, #51105; Qiagen) (26).

### Human Transcriptome Array

As previous reports have described (27), total mRNA samples from 18 healthy controls, 18 febrile controls, and 18 KD patients 24 hours prior to IVIG administration (KD1 group) and 18 KD patients 21 days after IVIG therapy (KD2 group) were pooled together into three RNA libraries, each containing RNA samples from six patients. All RNA samples were then prepared for hybridization to the GeneChip^®^ Human Transcriptome Array 2.0 (HTA 2.0, Affymetrix, Santa Clara) using the WT PLUS Reagent kit. Hybridized HTA 2.0 microarray chips were checked for quality, and the gene expression data was then analyzed with commercially available software (Partek, St. Louis).

### Confirmation of mRNA Expression With Reverse Transcription Polymerase Chain Reaction (RT-PCR)

We then confirmed leukocyte mRNA expression results of CD36 and AIM2 using RT-PCR. Total RNA obtained from leukocytes was first transformed into cDNA by following the manufacturer’s instructions (cDNA-High Capacity cDNA Reverse Transcription kit, Applied Biosystems, Cat. 4368813). We performed quantitative RT-PCR on the LightCycler R480 RT-PCR System (Roche Molecular System, USA) by adding 2.5 ng/μL of cDNA from each sample with 0.2μL (10μM) of forward and reverse primers and 5μL of SYBR Green Master Mix (ABI, Cat. No. 4367659). Relative mRNA expression levels were calculated by comparing the RT-PCR cycle number required to reach target fluorescence (the comparative threshold cycle method) using the equation 2^-(ΔCTtarget-ΔCTcalibrator)^, (i.e., 2^-ΔΔCT^). Relative mitochondrial DNA levels of the genes ND1, ND4, and COX1 that were present within plasma samples were also measured using SYBR Green dye-based quantitative PCR as described above. We conducted all experiments twice to verify amplification efficiency. The primers included in this study were as follows: 18S: (F) GTAACCCGTTGAACCCCATT, (R) CCATCCAATCGGTAGTAGCG; CD36: (F) CTCTTTCCTGCAGCCCAATG, (R) CTGCCACAGCCAGATTGAGA; AIM2: (F) TGGCCCAGCAGGAATCTATC, (R) TCTGTAGCCACTGTAGCATGA; ND1: (F) CCCTAAAACCCGCCACATCT, (R) GAGCGATGGTGAGAGCTAAGGT; ND4: (F) ACATCCTCATTACTATTCTG, (R) TTAGTGGGAGTAGAGTTT; COX1: (F) TCATCTGTAGGCTCATTC, (R) GGCATCCATATAGTCACT.

### Effect of Patient Plasma in U937-Derived Macrophages

We then tested the effect of patient plasma from healthy controls, febrile controls and patients with KD in U937-derived macrophages. U937 is a monocytic cell line that acquires macrophage-like properties after stimulation with phorbol 12-myristate 13-acetate (PMA, Sigma, # P8139-1) (28, 29). The U937 cell line was maintained in the Roswell Park Memorial Institute culture medium (RPMI 1640, ThermoFisher) containing 10% heat-inactivated fetal bovine serum (FBS, Invitrogen, # A1049101-01) and 1% Penicillin (GIBCO, #15140122). U937 was differentiated into macrophages by treating it with 200 μM PMA, followed by 48 h incubation in RPMI medium. Patient plasma from 10 healthy controls, 10 febrile controls and 20 patients with KD at a final concentration of 10% were then added into the medium for incubation for another 24 h. After incubation, total RNA was extracted using an RNA miniprep kit (ZYMO, #R2052). mRNA expression of CD36 and AIM2 were then determined by RT-PCR in the manner described in the previous paragraph.

### Statistical Analysis

Statistical analysis was performed using SPSS version 14.0 (SPSS, Inc., Chicago, USA). We compared data using one-way ANOVA for comparisons involving three groups or more and then adjusted *post-hoc* tests with Fisher’s least significant difference. Data comparison of two groups were compared using student’s *t*-test. All data are presented as mean ± standard error. All *p*-values less than 0.05 were considered statistically significant.

## Results

### Demographic Data

Demographic data of the 18 KD patients and 18 healthy and 18 febrile controls included for HTA 2.0 microarray analysis have been previously published elsewhere (17). Full microarray data has been uploaded to Pubmed and can be accessed *via* the NCBI GEO database (Series GSE109351). We also recruited an additional cohort of 52 healthy controls, 52 febrile controls, and 102 patients with KD, including 35 patients with coronary artery lesions, in a case control study. We observed no significant differences in terms of gender between the three groups. However, patients with KD were significantly younger than both the healthy and febrile controls (4.62 ± 0.59 vs. 3.07 ± 0.25, 1.80 ± 0.16 years of age, *p <*0.001, HC, FC and KD patients, respectively). Prior to IVIG therapy, patients with KD generally had higher white blood cell counts (8.77 ± 0.39 vs. 9.51 ± 0.62 vs. 13.87 ± 0.53 1000 cells/uL, *p <*0.001, HC, FC, and KD patients, respectively) and lower hemoglobulin levels (12.53 ± 0.13 vs. 12.17 ± 0.15 vs. 11.12 ± 0.09 mg/L *p <*0.001, HC, FC, and KD patients, respectively) than both the healthy and febrile controls. Of the 102 patients with KD, 35 patients (34.3%) had evidence of coronary artery lesions, and 10 patients (9.8%) had IVIG resistance (**Table 1**). While all KD patients in our study received low dose aspirin after they were discharged from the hospital, only 12 patients received high dose aspirin in the acute stage. In addition, only 2 of the patients in our study received methylprednisolone pulse, and none of the patients in our study received biological agents.

**Table 1:** Demographic characteristic of patients with KD and controls.

Characteristic	Healthy controls (n = 52)	Febrile controls (n =52)	Patients with KD (n = 102)	*p*-value
Male gender, n(%)	28 (53.8%)	30 (57.7%)	69 (67.6%)	0.198
Age (y)	4.62 ± 0.59	3.07 ± 0.25	1.80 ± 0.16	<0.001
Age range (y)	0-16	0-11	0-9	
WBC (1000/uL)	8.77 ± 0.39	9.51 ± 0.62	13.87 ± 0.53	<0.001
RBC (million/Ul)	4.85 ± 0.06	4.65 ± 0.07	4.29 ± 0.04	<0.001
Hemoglobin (g/dL)	12.53 ± 0.13	12.17 ± 0.15	11.12 ± 0.09	<0.001
CRP (mg/L)		27.67 ± 4.32	89.97 ± 6.89	<0.001
CAL formation			35 (34.3%)	
IVIG resistance			10 (9.8%)	

CAL, coronary artery lesion; IVIG, intravenous immunoglobulin; KD, Kawasaki disease.

data expressed as mean ± SEM.

### Patients With Acute KD Have Higher Levels of Circulating Cell Free Mitochondrial DNA in Plasma

**Figure 1:**
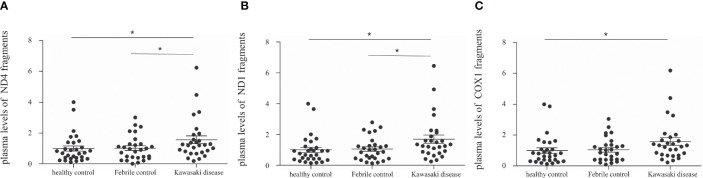
Comparison of cell-free mitochondrial DNA levels in the plasma of patients with Kawasaki disease (KD, n=29), healthy controls (HC, n=29), and febrile controls (FC, n=29). **(A)** Comparison of ND4 cell-free mitochondrial DNA (mean ± SEM, HC: 1 ± 0.17, FC: 1.02 ± 0.15, KD: 1.56 ± 0.25) **(B)** Comparison of ND1 cell-free mitochondrial DNA (mean ± SEM, HC: 1 ± 0.17, FC: 1.06 ± 0.14, KD: 1.69 ± 0.26) **(C)** Comparison of COX1 cell-free mitochondrial DNA (mean ± SEM, HC: 1 ± 0.18, FC: 1.11 ± 0.15, KD: 1.58 ± 0.25). An asterisk denotes a p-value of less than 0.05.

Circulating cell-free mitochondrial DNA was first extracted from the plasma DNA of patients with acute KD prior to IVIG therapy and healthy and febrile controls. We then used quantitative PCR of the three mitochondrial genes ND1, ND4, and COX1 to determine the amount of plasma mitochondrial DNA present in the plasma samples. We found that patients with acute Kawasaki disease had statistically significant higher levels of ND1, ND4, and COX1 mitochondrial DNA when compared to healthy controls (relative gene expression KD vs. HC: 1.69 ± 026 vs. 1 ± 0.17, 1.56 ± 0.25 vs. 1 ± 0.17, 1.58 ± 0.25 vs. 1 ± 0.18, *p* = 0.016, 0.047, 0.042 for ND1, ND4, and COX1, respectively) and febrile controls (relative gene expression KD vs. FC: 1.69 ± 0.26 vs. 1.06 ± 0.14, 1.56 ± 0.25 vs. 1.02 ± 0.15, 1.58 ± 0.25 vs. 1.11 ± 0.15, *p* = 0.028, 0.043, 0.095 for ND1, ND4, and COX1, respectively) (**Figure 1**).

### CD36 Is Overexpressed in Patients With KD and Is Associated With Coronary Artery Lesions

**Figure 2:**
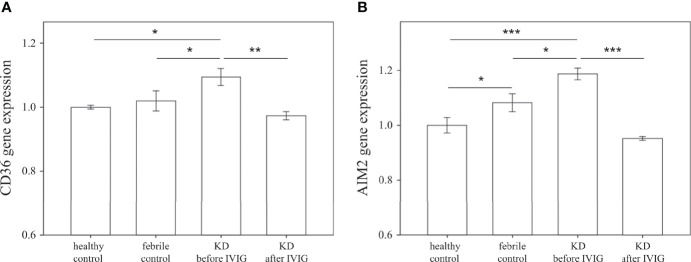
Comparison of relative mRNA expression levels on HTA 2.0 microarray between patients with Kawasaki disease before IVIG (KD1, n=18), after IVIG (KD2, n=18), healthy controls (HC, n=18), and febrile controls (FC, n=18). **(A)** Comparison of relative CD36 mRNA expression levels (mean ± SEM, HC: 1 ± 0.01, FC: 1.02 ± 0.03, KD1: 1.09 ± 0.03, KD2: 0.97 ± 0.01) **(B)** Comparison of relative AIM mRNA expression levels (mean ± SEM, HC: 1 ± 0.03, FC: 1.08 ± 0.03, KD1: 1.19 ± 0.02, KD2: 0.95 ± 0.01). An asterisk denotes a p-value of less than 0.05.

**Figure 3:**
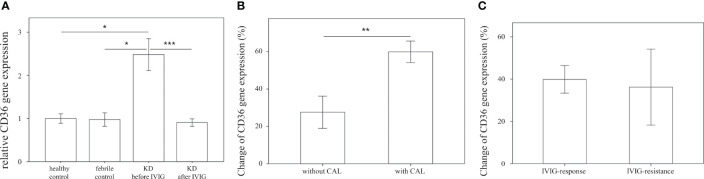
Comparison of relative CD36 mRNA expression. **(A)** Relative CD36 mRNA expression levels in healthy controls (n=23, mean ± SEM 1 ± 0.11), febrile controls (n=24, mean ± SEM 0.97 ± 0.16), and in Kawasaki disease patients before IVIG (n=71, mean ± SEM 2.48 ± 0.37) and after IVIG (n=72, mean ± SEM 0.91 ± 0.09). **(B)** Comparison of relative CD36 percent changes before and after IVIG therapy in KD patients without CAL (n=46, mean ± SEM 0.28 ± 0.09) and with CAL (n=26, mean ± SEM 0.60 ± 0.06). Percentage changes were calculated *via* the following equation (KD1-KD2)/KD1 * 100%. **(C)** Comparison of relative CD36 percent changes before and after IVIG therapy in KD patients with adequate IVIG response (n=63, mean ± SEM 0.40 ± 0.07) and with IVIG resistance (n=9, mean ± SEM 0.36 ± 0.18). Percentage changes were calculated using the following equation (KD1-KD2)/KD1 * 100%. An asterisk denotes a p-value of less than 0.05.

We first examined the mRNA expression of CD36 on HTA 2.0 microarray and found that CD36 expression in patients with acute KD was significantly higher than that in both healthy (relative gene expression KD1 vs. HC 1.09 ± 0.03 vs. 1 ± 0.01, *p* = 0.015) and febrile controls (relative gene expression KD1 vs. FC 1.09 ± 0.03 vs. 1.02 ± 0.03, *p* = 0.040). Furthermore, CD36 mRNA expression decreased significantly after IVIG therapy (relative gene expression KD1 vs. KD2 1.09 ± 0.03 vs. 0.97 ± 0.01, *p* = 0.004) (**Figure 2A**). We then confirmed CD36 mRNA expression in a separate cohort of patients using quantitative PCR and found that CD36 expression was significantly higher in acute KD patients when compared to both healthy (relative gene expression KD1 vs. HC 2.48 ± 0.37 vs. 1 ± 0.11, *p* = 0.015) and febrile controls (relative gene expression KD1 vs. FC 2.48 ± 0.37 vs. 0.97 ± 0.16, *p* = 0.011). Similarly, CD36 mRNA expression decreased significantly following IVIG therapy (relative gene expression KD1 vs. KD2 2.48 ± 0.37 vs. 0.91 ± 0.09 *p* < 0.001) (**Figure 3A**). We also found that KD patients with coronary artery lesions had a higher percentage decrease of CD36 mRNA expression when compared to those without coronary artery lesions (relative gene expression 0.60 ± 0.06% vs. 0.28 ± 0.09%, *p* = 0.003), but this finding was not associated with IVIG resistance (**Figures 3B, C**). In patients who had received high dose aspirin or methylprednisolone pulse therapy, expression levels of CD36 were not significantly different from those who did not, either before or after IVIG therapy (**Supplemental Table 1**).

### AIM2 Is Overexpressed in Patients With KD and Is Associated With Coronary Artery Lesions

**Figure 4:**
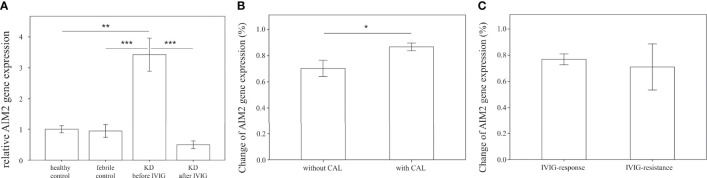
Comparison of relative AIM2 mRNA expression. **(A)** Relative AIM2 mRNA expression levels in healthy controls (n=23, mean ± SEM 1 ± 0.11), febrile controls (n=24, mean ± SEM 0.94 ± 0.21), and in Kawasaki disease patients before IVIG (n=71, mean ± SEM 3.43 ± 0.53) and after IVIG (n=71, mean ± SEM 0.50 ± 0.12). **(B)** Comparison of relative AIM2 percent changes before and after IVIG therapy in KD patients without CAL (n=46, mean ± SEM 0.70 ± 0.06) and with CAL (n=25, mean ± SEM 0.87 ± 0.03). Percentage changes were calculated using the following equation (KD1-KD2)/KD1 * 100%. **(C)** Comparison of relative CD36 percent changes before and after IVIG therapy in KD patients with adequate IVIG response (n=62, mean ± SEM 0.77 ± 0.04) and with IVIG resistance (n=9, mean ± SEM 0.71 ± 0.18). Percentage changes were calculated using the following equation (KD1-KD2)/KD1 * 100%. An asterisk denotes a p-value of less than 0.05. .

We further examined the mRNA expression of AIM2 on HTA 2.0 microarray and found that AIM2 expression in patients with acute KD was significantly higher than that of both healthy (relative gene expression KD1 vs. HC 1.19 ± 0.02 vs. 1 ± 0.03, *p* < 0.001) and febrile controls (relative gene expression KD1 vs. FC 1.19 ± 0.02 vs. 1.08 ± 0.03, *p* = 0.016); it also decreased significantly after IVIG therapy (relative gene expression KD1 vs. KD2 1.19 ± 0.02 vs. 0.95 ± 0.01, *p* < 0.001) (**Figure 2B**). The mRNA expression of AIM2 was then confirmed in a separate cohort of patients. Results from quantitative PCR replicated the findings found on HTA 2.0 microarray. Again, AIM2 mRNA expression was significantly higher in patients with acute KD when compared to both the healthy (relative gene expression KD1 vs. HC 3.43 ± 0.53 vs. 1 ± 0.11, *p* = 0.001) and febrile controls (relative gene expression KD1 vs. FC 3.43 ± 0.53 vs. 0.94 ± 0.21, *p* < 0.001). AIM2 mRNA expression also decreased significantly after IVIG therapy (relative gene expression KD1 vs. KD2 3.43 ± 0.53 vs. 0.50 ± 0.12, *p* < 0.001) (**Figure 4A**). We also found that KD patients who developed coronary artery lesions had a greater decrease in AIM2 mRNA expression after IVIG therapy (relative gene expression 0.87 ± 0.03% vs. 0.70 ± 0.06%, p = 0.020), although changes in AIM2 mRNA expression were not associated with IVIG resistance (**Figures 4B, C**).

### KD Patient Plasma Induces Increased Expression of CD36 and AIM2 in U937-Derived Macrophage Cells

**Figure 5:**
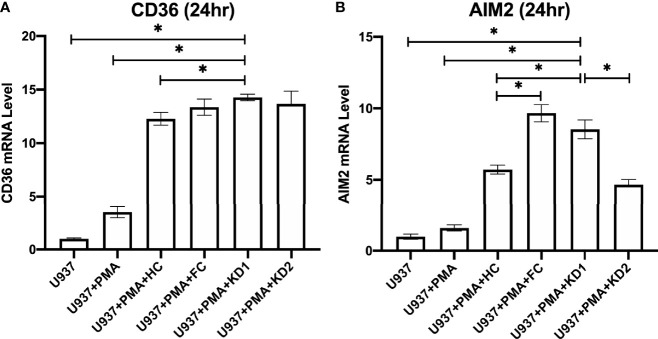
Comparison of relative CD36 and AIM2 mRNA expression in U937-derived macrophages after stimulation with patient plasma. **(A)** Relative CD36 mRNA expression levels in U937 cell cultures (n=3, mean ± SEM 1 ± 0.08), U937 cells stimulated with PMA (n=3, mean ± SEM 3.49 ± 0.52), U937 cells stimulated with PMA and plasma from healthy patients (n=10, mean ± SEM 12.27 ± 0.59), U937 cells stimulated with PMA and plasma from KD patients 24 hours prior to IVIG therapy (n=10, mean ± SEM 14.26 ± 0.31), and U937 cells stimulated with PMA and plasma from KD patients 21 days after IVIG therapy (n=10, mean ± SEM 13.66 ± 1.19). **(B)** Relative AIM2 mRNA expression levels in U937 cell cultures (n=3, mean ± SEM 1 ± 0.18), U937 cells stimulated with PMA (n=3, mean ± SEM 1.60 ± 0.22), U937 cells stimulated with PMA and plasma from healthy patients (n=10, mean ± SEM 5.72 ± 0.31), U937 cells stimulated with PMA and plasma from KD patients 24 hours prior to IVIG therapy (n=10, mean ± SEM 8.55 ± 0.65), and U937 cells stimulated with PMA and plasma from KD patients and 21 days after IVIG therapy (n=10, mean ± SEM 4.67 ± 0.36). An asterisk denotes a p-value of less than 0.05. HC, Healthy control; KD1, KD prior to IVIG; KD2, KD after IVIG.

In order to examine the changes in CD36 and AIM2 expression specifically in macrophages, we added patient plasma at a final concentration of 10% from KD patients and controls to U937-derived macrophages. U937 is a monocytic cell line that transforms into macrophage-like cells after stimulation with PMA. U937-derived macrophages were stimulated with patient plasma from healthy controls, febrile controls and from patients with KD prior to IVIG treatment and 21 days after IVIG treatment for 24 hours. We found that U937-derived macrophages which were stimulated with plasma from the febrile controls had similar mRNA expression levels of CD36 when compared to healthy controls (FC Vs. HC, Mean ± SE: 13.36 ± 0.76 Vs. 12.22 ± 0.60, p = 0.328) (**Figure 5A**), but significantly higher AIM2 mRNA expression levels (FC Vs. HC, Mean ± SE: 9.67 ± 0.60 Vs. 5.72 ± 0.31, p < 0.001) (**Figure 5B**).

We also found that AIM2 mRNA expression was significantly higher in U937-derived macrophages that had been stimulated with plasma from patients with acute KD when compared to the U937-derived macrophages that had been stimulated with plasma from healthy controls (8.55 ± 0.65 vs. 5.72 ± 0.31, *p* < 0.001, **Figure 5B**). Stimulation with plasma from patients with acute KD also increased mRNA expression of CD36 in U937-derived macrophages, although this increase did not reach statistical significance when compared to stimulation with plasma from healthy controls (**Figure 5A**).

## Discussion

The effective clearance of apoptotic cells by macrophages and dendritic cells is important for maintaining immune homeostasis and developing peripheral tolerance (30). Macrophages express scavenger receptors, including SCARF1, CD305, CD11b, CD11c, and CD36, which aid in the phagocytosis of apoptotic cells and cellular debris (30). In particular, CD36 mediates the interaction of macrophages and apoptotic cellular debris by binding to oxidized phosphatidylserine, which is typically expressed on the inner side of the plasma membrane but is exposed in apoptotic cells, which is considered an important “eat me” signal (31). CD36 also contains high-affinity binding sites for thrombospondin-1 (TSP-1) (32), which is secreted by apoptotic cells and induces the transformation of dendritic cells into an immature dendritic cell tolerogenic state, thus promoting self-tolerance (33).

In contrast, the ineffective clearance of apoptotic cellular debris leads to exposure of autoantigens from within the apoptotic cell body, leading to the production of autoantibodies, the loss of self-tolerance, and increased inflammation. Impaired clearance of apoptotic cells has been linked to several autoimmune diseases, such as systemic lupus erythematosus, primary biliary cirrhosis, and autoimmune lymphoproliferative syndrome (34). Plasma mitochondrial DNA are also secreted by apoptotic cells (20) and are regarded as a damage-associated molecular pattern (DAMP) that can stimulate pro-inflammatory responses *via* toll-like receptor 9 (TLR9) and activate the inflammasome pathway (19).Inflammasome activation within macrophages is believed to occur in two steps. First, microparticles including mitochondrial DNA released from damaged cells enter the cell *via* endocytosis and bind to TLR9, increasing transcription of proinflammatory genes pro-IL-1β and pro IL-18 *via* activation of the transcription factor NF- kb (signal 1). Cellular stress can also trigger intracellular mitochondrial damage and the release of oxidized mitochondrial DNA into the cytosol, which then initiates the aggregation of inflammasome receptor proteins like NLRP3, NLRC4, and AIM2 and the activation of caspase 1, which cleaves pro-IL-1β and pro-IL-18 into their mature secreted forms of IL-1β and IL-18 (signal 2), respectively (19, 35).

Although the exact immunopathogenesis of KD is not completely understood, evidence has been found in human patients and both *in vitro* and *in vivo* disease models that show that inflammasome activation is one of the many important inflammatory pathways involved in its development. In our previous research which examined the expression levels of three NLRC genes, 14 NLRP genes and the two primary cytokine products of the inflammasome pathway IL-18 and IL-1b, we found that patients with KD had increased mRNA expression of NLRC4, NLRP12, and IL-1β mRNA levels (36). Other studies have also found that plasma levels of inflammasome related proteins, including caspase-1, IL-1β, and IL-18, were elevated in the plasma of KD patients when compared to healthy controls. Evidence of inflammasome involvement has also been found in both *in vivo* and *in vitro* models of KD. KD-sera treated monocytic cells (THP1) were able to induce increased expression and activation of such pyroptosis proteins as caspase-1, IL-1β, and IL-18 in human umbilical vein endothelial cells (37). In a Candida albicans water-soluble fraction (CAWS) induced mouse model of KD, NLRP3 inflammation activation was crucial to the development of coronary artery lesions and could be circumvented by IL-1β, NLRP3, and ASC depletion (38).

There are several limitations to our manuscript. For one, we used mRNA obtained from total leukocytes to examine the mRNA expression of CD36 within KD patients, when ideally it would have been best to examine the expression of CD 36 within isolated peripheral monocytes. In practice, it was very difficult to draw large enough blood samples in our young KD patients to obtain a sufficient number of monocytes for analysis, as monocytes make up only 5-10% of the total leukocyte population. In spite of this it should be noted that although CD36 is indeed expressed on many cell types, it is most abundantly expressed in monocytes and can be used as a monocyte cell marker (39). We have tried to work around this problem by reconfirming our findings in a U937 monocyte cell model, and found that stimulation with KD patient plasma obtained prior to IVIG resulted in higher CD36 expression when compared to stimulation with KD patient plasma obtained after IVIG. It is also possible that changes in CD36 mRNA expression before and after IVIG therapy may also reflect changes in leukocyte percentages, as one recent study found that IVIG treatment led to reductions in monocyte numbers after IVIG treatment (40). Nonetheless, we found that the percentage change in CD36 expression before and after IVIG therapy was not associated with changes in the percentage of segmented neutrophils, lymphocytes, monocytes or eosinophils (**Supplemental Table 2**). Therefore, we tentatively conclude that changes in CD36 expression before and after IVIG therapy are quite possibly the result of decreased expression, and do not seem to be associated with changes in leukocyte cell subpopulations.

Another limitation of this study is that data regarding the lipid profile of the samples could were not obtained for analysis. There is reason to believe that high plasma lipids would contribute to the increased expression of CD36. As an example, mice fed high fat diets do indeed have increased expression of CD36 within the liver (14). However, a recent meta-analysis including a total of 1684 subjects (990 KD patients and 694 controls) found that patients with KD had no significant differences in triglycerides, total cholesterol or low-density lipoprotein cholesterol when compared with controls (41). Because lipid levels were not tested in the subjects in our study, it is difficult to say whether changes in CD36 expression were associated with changes in plasma lipid levels.

In this study, we found that patients with acute Kawasaki disease had higher plasma levels of cell free mitochondrial DNA (ND1, ND4, and COX1) when compared to both healthy and febrile controls, which decreased after IVIG therapy. Similarly, patients with KD also had higher mRNA expression of the macrophage scavenger receptor CD36 and the inflammasome receptor AIM2 when compared to healthy and febrile controls, both of which were reduced following treatment with IVIG. We also found that a greater percent decrease in both CD36 and AIM2 mRNA expression 21 days after IVIG therapy was associated with the development of coronary artery lesion but not with IVIG resistance in patients with KD. Altogether, our findings suggest that KD patients in the acute phase may have a higher burden of apoptotic cellular debris, higher expression of CD36, a scavenger receptor that aids in the clearance of apoptotic debris, and higher expression of AIM2, which may induce subsequent involvement of the inflammasome pathway. Moreover, patients with coronary artery lesions had a larger percentage decrease of CD36 expression 21 days after IVIG therapy, suggesting prolonged expression of the scavenger receptor, and thus prolonged apoptotic debris clearance may have a protective effect against the development of coronary artery lesions. Further studies are needed to clarify the possible mechanistic link between CD36 and the development of Kawasaki disease.

## Data Availability Statement

The datasets presented in this study can be found in online repositories. The names of the repository/repositories and accession number(s) can be found below: https://www.ncbi.nlm.nih.gov/, GEO: Series GSE109351.

## Ethics Statement

Informed consent was obtained from the parents or guardians of all patients included in this study. The studies involving human participants were reviewed and approved by the Internal Review Board of Chang Gung Memorial Hospital.

## Author Contributions

Y-HH, H-CK, MM-HG, and F-SW contributed to conception and design of the study. MM-HG, L-SC, Y-HH, and H-CK performed data curation, laboratory work, cell culture and data organization. MM-HG, Y-HH, and K-DC performed the statistical analysis. MM-HG wrote the first draft of the manuscript. Y-HH wrote sections of the manuscript. All authors contributed to manuscript revision, read, and approved the submitted version.

## Funding

This study received funding from the Ministry of Science and Technology MOST 108-2314-B-182 -037 -MY3 and grants provided by Chang Gung Memorial Hospital (CMRPG8J0321, CMRPG8J1151, CMRPG8L0031, CMRPG8L0021). The funders had no role in study design, collection, analysis, and interpretation of data, writing or the decision to submit this paper for publication.

## Conflict of Interest

The authors declare that the research was conducted in the absence of any commercial or financial relationships that could be construed as a potential conflict of interest.

## Publisher’s Note

All claims expressed in this article are solely those of the authors and do not necessarily represent those of their affiliated organizations, or those of the publisher, the editors and the reviewers. Any product that may be evaluated in this article, or claim that may be made by its manufacturer, is not guaranteed or endorsed by the publisher.
