# Supplementary material for: CD36 is Associated With the Development of Coronary Artery Lesions in Patients With Kawasaki Disease
Source: Front Immunol. 2022 Jan 27;13:790095. doi: 10.3389/fimmu.2022.790095 (PMC8828496; doi:10.3389/fimmu.2022.790095)
Supplement: Supplementary file 1 [file DataSheet_1.docx]

Supplemental Data

Table 1: Comparison of CD36 relative mRNA expression in KD patients

|  | No high dose aspirin | High dose aspirin | *p-*value |
| --- | --- | --- | --- |
| Relative CD36 mRNA expression before IVIG | 2.47 ± 0.40  (n=60) | 2.58 ± 1.00  (n=12) | 0.908 |
| Relative CD36 mRNA expression after IVIG | 0.83 ± 0.10  (n=59) | 1.17 ± 0.11  (n=12) | 0.157 |
|  | No MP pulse | MP pulse | *p-*value |
| Relative CD36 mRNA expression before IVIG | 2.53 ± 0.38  (n=70) | 1.00 ± 0.00  (n=2) | 0.504 |
| Relative CD36 mRNA expression after IVIG | 0.90 ± 0.09  (n=69) | 0.50 ± 0.50  (n=2) | 0.461 |

**Data is expressed as mean ± standard error

Table 2: Correlation between percentage decrease in CD36 mRNA expression and percentage decrease in leukocyte subpopulations before and after IVIG therapy

|  | Pearson’s Correlation | p-value |
| --- | --- | --- |
| Segmented Neutrophils | 0.042 | 0.723 |
| Lymphocytes | -0.055 | 0.644 |
| Monocytes | 0.051 | 0.070 |
| Eosinophils | 0.007 | 0.953 |
| Basophils | 0.079 | 0.512 |
